# Relationship of psychotropic medication use with physical function among postmenopausal women

**DOI:** 10.1007/s11357-024-01141-z

**Published:** 2024-03-22

**Authors:** Hind A. Beydoun, May A. Beydoun, Edward Kwon, Brook T. Alemu, Alan B. Zonderman, Robert Brunner

**Affiliations:** 1 Department of Research Programs, A.T. Augusta Military Medical Center, 9300 DeWitt Loop, Fort Belvoir, VA 22060 USA; 2 Laboratory of Epidemiology and Population Sciences, National Institute on Aging, NIA/NIH/IRP, Baltimore, MD USA; 3 Department of Family Medicine, A.T. Augusta Military Medical Center, 9300 DeWitt Loop, Fort Belvoir, VA 22060 USA; 4 Health Sciences Program, School of Health Sciences, Western Carolina University, Cullowhee, NC USA; 5 Department of Family and Community Medicine (Emeritus), School of Medicine, University of Nevada Reno, Reno, NV USA

**Keywords:** Antidepressant, Anxiolytic, Hypnotic, Physical function, Psychotropic, Sedative

## Abstract

To examine cross-sectional and longitudinal relationships of psychotropic medications with physical function after menopause. Analyses involved 4557 Women’s Health Initiative Long Life Study (WHI-LLS) participants (mean age at WHI enrollment (1993–1998): 62.8 years). Antidepressant, anxiolytic, and sedative/hypnotic medications were evaluated at WHI enrollment and 3-year follow-up visits. Performance-based physical function [Short Physical Performance Battery (SPPB)] was assessed at the 2012–2013 WHI-LLS visit. Self-reported physical function [RAND-36] was examined at WHI enrollment and the last available follow-up visit—an average of 22 [±2.8] (range: 12–27) years post-enrollment. Multivariable regression models controlled for socio-demographic, lifestyle, and health characteristics. Anxiolytics were not related to physical function. At WHI enrollment, antidepressant use was cross-sectionally related to worse self-reported physical function defined as a continuous (*β* = −6.27, 95% confidence interval [CI]: −8.48, −4.07) or as a categorical (< 78 vs. ≥ 78) (odds ratio [OR] = 2.10, 95% CI: 1.48, 2.98) outcome. Antidepressant use at WHI enrollment was also associated with worse performance-based physical function (SPPB) [< 10 vs. ≥ 10] (OR = 1.53, 95% CI: 1.05, 2.21) at the 2012–2013 WHI-LLS visit. Compared to non-users, those using sedative/hypnotics at WHI enrollment but not at the 3-year follow-up visit reported a faster decline in physical function between WHI enrollment and follow-up visits. Among postmenopausal women, antidepressant use was cross-sectionally related to worse self-reported physical function, and with worse performance-based physical function after > 20 years of follow-up. Complex relationships found for hypnotic/sedatives were unexpected and necessitate further investigation.

**Supplementary Information:**

The online version contains supplementary material available at 10.1007/s11357-024-01141-z.

## Introduction

The decline in physical functioning that accompanies aging has detrimental implications for quality of life [1], well-being [2], and independence [3]. Epidemiologic studies of older adults suggest that self-reported and performance-based physical function may be worsened by frequently co-occurring depression [4–11], anxiety [5, 10, 12–14], and sleep disorders [13, 15–27]. The impact of anxiety and depression on physical function appears to be complex. For instance, a study of older patients with knee osteoarthritis suggested that while higher anxiety was related to poorer self-reported physical function, neither anxiety nor depression was related to performance-based physical function [9]. By contrast, among community-dwelling adults, ≥ 80 years of age, poor sleep efficiency and frequent waking after sleep onset were negatively associated with walking speed and knee extension strength [21]. A gap exists as to how psychotropic (antidepressants [28–30], anxiolytic [12], and sedative/hypnotic [12, 31, 32]) medications that were used to treat depression, anxiety, and sleep disorders may alter physical function. One issue is that psychotropic medications are used for a variety of indications. Nevertheless, they are known to increase the risk of falls and fractures, to slow gait speed, and to promote neurocognitive dysfunction in older populations [33–37]. Antidepressant use has been specifically evaluated in relation to morbidity and mortality risks among postmenopausal women [33–37], with much less known about the health implications of anxiolytic [38] and sedative/hypnotic [38, 39] use after menopause. There is limited evidence linking any of the psychotropic medications to physical function among older adults [28–30, 40], in general, and in postmenopausal women [32], in particular. Most available studies have ≤ 10 years of follow-up and assessed psychotropic medication use and physical function at a single time point. The Women’s Health Initiative (WHI) study, and specifically, the WHI Long Life Study (WHI-LLS), allows us to examine cross-sectional and longitudinal relationships of antidepressant, anxiolytic, and sedative/hypnotic medication use to physical function among postmenopausal women with > 20 years of follow-up. Use of antidepressants, anxiolytics, and sedative/hypnotics may be associated with poorer physical function not only because they are considered a *proxy* for underlying depression, anxiety, and sleep disorders but also because they may reflect level of disease severity and can result in side-effects that are detrimental to physical function [41]. In this study, we hypothesized that psychotropic medication use is related to poorer physical function at baseline and over time among postmenopausal women.

## Methods

### Data sources

The WHI is a long-term study focused on strategies for preventing heart disease, breast, and colorectal cancers as well as osteoporosis in postmenopausal women. The WHI study design, eligibility criteria, recruitment methods, and measurement protocols are described elsewhere [42, 43]. Briefly, the WHI collected data on a multiethnic sample of postmenopausal women who were recruited and enrolled between 1993 and 1998 at 40 geographically diverse clinical centers (24 states and the District of Columbia) in the United States. The WHI study received institutional review board approval with informed consent from all participating clinical centers. WHI-Clinical Trials (*n* = 68,132) and the WHI-Observational Study (*n* = 93,676) are two components of the WHI with a combined enrollment of 161,808 participants. Whereas WHI-CTs evaluated outcomes of menopausal hormone therapy (Hormone Therapy [HT] Trials), calcium and vitamin D supplementation ([CaD] Trial), and a low-fat eating pattern (Dietary Modification Trial), the WHI-OS evaluated causes of morbidity and mortality in postmenopausal women. At enrollment (1993–1998), WHI participants, 50–79 years of age, completed the same self-administered questionnaire covering demographics, general health, clinical and anthropometric characteristics, functional status, healthcare behaviors, reproductive, medical, and family history, personal habits, thoughts and feelings, therapeutic class of medication, hormones, supplements, and dietary intake, and several of these characteristics were assessed at later follow-up times. The main analyses for this paper were performed using data on WHI participants who enrolled in the WHI-LLS which was conducted in 2012–2013. The WHI-LLS involves a one-time, in-person, visit for collecting phlebotomy, anthropometry, and objective physical function measures in a subset of WHI participants. The WHI-LLS included former participants of the WHI HT, as well as African American and Hispanic women. Women who resided in an institution or were unable to provide informed consent due to dementia were excluded from the WHI-LLS. Of 14,081 WHI participants who were eligible for the WHI-LLS, 7875 women, 63–99 years of age, successfully completed at-home visits between March 2012 and May 2013 [44–46]. An additional sub-cohort of WHI-CT participants (≥ 65 years of age at enrollment) had performance-based physical function tests at enrollment (1993–1998), 1 year, 3 years, and 6 years follow-up visits [47]. This sub-cohort provided the opportunity to conduct more detailed longitudinal analyses of hypothesized relationships between psychotropic medications and physical function. This project was determined as exempt research by the Institutional Review Board of the National Institute on Aging.

### Study variables

#### Psychotropic medications

WHI participants were instructed to bring prescription and non-prescription medication containers at enrollment (1993–1998) and 3-year follow-up visits. With respect to medications used for > 2 weeks, drug names and doses were entered into a medications database and assigned therapeutic class codes using the Master Drug Data Base (MDDB: Medi-Span, Indianapolis, IN; Medi-Span software: First DataBank, Inc., San Bruno, CA). A series of dichotomous (“yes” or “no”) variables were defined for psychotropic (antidepressants, anxiolytics, sedative/hypnotics) medication use, at baseline and 3 years of follow-up, based on therapeutic class codes (See ESM 1 Appendix Methods for details regarding specific medications).

#### Patterns of psychotropic medication use

Taking non-users of psychotropic medications as a referent category, we defined a variable that combines the dichotomous (“yes” or “no”) variables for antidepressants, anxiolytics, and sedative/hypnotics at WHI enrollment (1993–1998). Specifically, an interaction variable was defined as follows: *antidepressant only*, *anxiolytic only*, *sedative/hypnotics only*, *antidepressant + anxiolytic*, *antidepressant + sedative/hypnotics*, *anxiolytic + sedative/hypnotics*, *antidepressant + anxiolytic + sedative/hypnotics*. Taking sample size limitations into account, this variable was later categorized as *antidepressant only*, *anxiolytic only*, *sedative/hypnotic only*, and *combined* psychotropic medication use. For each type of psychotropic medication, we defined patterns of use between enrollment (1993–1998) and 3-year follow-up visits as *non-user*, *user at enrollment only*, *user at follow-up only*, or *user at enrollment and follow-up visits* (See ESM 1 Appendix Methods for details regarding sample sizes).

#### Self-reported physical function

The RAND-36 Item Survey was used to assess repeated measures of self-reported physical function at WHI enrollment (1993–1998) and subsequent follow-up visits. It consists of 10 survey questions regarding self-perceived difficulty in specific functional activities [moderate/vigorous activity (2 items); strength to lift, carry, stoop, bend, stair climb (4 items); ability to walk various distances without difficulty (3 items); and self-care (1 item)], each scored as “1=a lot,” “2=a little,” or “3=not at all”, and transformed to generate a total score of 0–100, with higher scores reflecting better function. The RAND-36 was initially administered to WHI-CT participants at enrollment (1993–1998), year 1 and end of follow-up and to WHI-OS participants at enrollment (1993–1998) and year 3 of follow-up, and subsequently at later follow-up times. Continuous RAND-36 scores were dichotomized as < 78 indicating low function or ≥ 78 indicating high function [22, 48–58]. Change in RAND-36 score over time was also calculated by dividing the difference in RAND-36 score between the last available follow-up and enrollment (1993–1998) visits by the age difference between these two visits. The latest data whereby RAND-36 items were administered using Form 521 became available on February 19, 2022, with durations of follow-up for WHI participants ranging between 7 and 28 years (See ESM 1 Appendix Methods for details regarding sample sizes).

#### Performance-based physical function

Trained research staff conducted in-person evaluations of grip strength and tested components of the Short Physical Performance Battery (SPPB) [balance, timed walk, and chair stand tests] at the 2012–2013 WHI-LLS home visit, with mean [±SD] of 16.0 [±1.2] (range: 14–19) years post-WHI enrollment (1993–1998). Accordingly, we defined performance-based physical function at the 2012–2013 WHI-LLS home visit using scores that combined these measurements, ranging between 0 and 12, with a score of < 10 indicating worse physical function [22, 48–50, 52–58]. Because SPPB evaluations were not consistently available at WHI enrollment (1993–1998) or earlier follow-up visits among the 2012–2013 WHI-LLS cohort, and because of the long period of time between enrollment (1993–1998) and 2012–2013 WHI-LLS visits, we also examined performance-based physical function over time using data on 5985 WHI-CT participants (≥ 65 years of age at enrollment) for whom specific SPPB evaluations (grip strength, chair stands, gait speed) were performed at WHI enrollment (1993–1998), 1 year, 3 years, and 6 years follow-up visits [47] (See ESM 1 Appendix Methods for details regarding sample sizes).

#### Covariates

We evaluated several characteristics collected at the WHI enrollment (1993–1998) visit as potential confounders of hypothesized relationships. These characteristics, which are known to be associated with the exposure (psychotropic medications) and outcome (physical function) variables of interest, and are not on the causal pathway between them, include WHI component, *socio-demographic* characteristics (age, race, ethnicity, education, household income, marital status), *lifestyle* characteristics (smoking status, alcohol consumption, physical activity), and *health* characteristics, namely, body mass index (BMI), comorbid conditions (cardiovascular disease, hypertension, hyperlipidemia, diabetes), symptoms of depression and insomnia, as well as self-rated health. Trained staff collected anthropometric data, including weight [kg] and height [cm] at enrollment [59], and BMI was calculated as (weight (kg) ÷ (height^2^ (m^2^)) and further categorized as < 25.0 kg/m^2^ [underweight/normal weight]; 25.0–29.9 kg/m^2^ [overweight]; and ≥ 30 kg/m^2^ [obese]. History of cardiovascular disease was defined in terms of previous coronary heart disease, angina, aortic aneurysm, carotid endarterectomy or angioplasty, atrial fibrillation, congestive heart failure, cardiac arrest, stroke, or transient ischemic attack. History of hypertension was defined as a self-reported diagnosis or treatment for hypertension or evidence of high blood pressure based on systolic and diastolic blood pressure measurements. History of diabetes was defined as physician-diagnosed diabetes or use of diabetes medications. History of hyperlipidemia was defined as using lipid-lowering medications or having been told of high cholesterol by a physician. A depressive symptoms screening algorithm previously developed by Burnam *et al.* with scores ranging between 0 and 1 and higher scores consistent with a greater burden of depressive symptoms was generated using 6 items from the 20-item Center for Epidemiologic Studies Depression Scale (CES-D) and 2 items from the National Institute of Mental Health’s Diagnostic Interview Schedule (DIS). Furthermore, we dichotomized this variable based on a pre-established threshold of 0.06, whereby WHI participants with a score > 0.06 have strong evidence of depressive symptoms while those with a score ≤ 0.06 did not [60–64]. The WHI Insomnia Rating Scale (WHIIRS) consists of five items: whether participants had trouble falling asleep, woke up several times at night, woke up earlier than planned, and had trouble getting back to sleep after awakening early over the past 4 weeks (*i.e.*, response categories coded as follows: 0 = “no, not in the past 4 weeks”; 1 = “yes, less than once per week;” 2 = “yes, 1 to 2 times per week;” 3 = “yes, 3 or 4 times per week;” and 4 = “yes, 5 or more times per week,”) as well as one item on overall sleep quality (coded as: 0 = “very sound or restful,” 1 = “sound or restful,” 2 = “average quality,” 3 = “restless,” and 4 = “very restless.”) These items were summed to calculate an overall insomnia score (range: 0–20). WHIIRS scores > 9 were consistent with diagnostic criteria for insomnia [60, 65–76].

### Statistical analyses

All statistical analyses were conducted using SAS version 9.4 (SAS Institute, Cary, NC). Summary statistics included mean ± standard deviation (SD) for continuous variables and frequencies with percentages for categorical variables. Bivariate associations were examined using the chi-square test, independent samples *t*-test, Pearson’s correlation coefficient, or their non-parametric counterparts, as appropriate. Linear and logistic regression models were constructed to estimate *β* coefficients or odds ratios (OR) with their 95% confidence intervals (CI), before and after controlling for confounders. Linear regression models were applied when the outcome was defined as a continuous variable, whereas logistic regression models were applied when self-reported physical function was defined as < 78 versus ≥ 78 or performance-based physical function was defined as < 10 versus ≥ 10. First, we examined psychotropic (antidepressant, anxiolytic, sedative/hypnotic) medication use in relation to demographic, socioeconomic, lifestyle, and health characteristics at WHI enrollment (1993–1998). Second, we examined self-reported physical function (at enrollment and annualized change between visits) as well as performance-based physical function at the 2012–2013 WHI-LLS visit in relation to demographic, socioeconomic, lifestyle, and health characteristics at WHI enrollment (1993–1998). Finally, we examined cross-sectional and longitudinal relationships of psychotropic medication use with physical function scores, before and after controlling for demographic, socioeconomic, lifestyle, and health characteristics measured at WHI enrollment (1993–1998). Specifically, we constructed fixed-effects and mixed-effects linear and logistic regression models to evaluate antidepressant, anxiolytic, and sedative/hypnotic medication use and their combinations (patterns of use) at WHI enrollment (1993–1998) in relation to (1) self-reported physical function at WHI enrollment (1993–1998), (2) change in self-reported physical function between WHI enrollment (1993–1998) and last available follow-up visits, and (3) performance-based physical function (*2012–2013 WHI-LLS* visit; *WHI-CT random sample:* WHI enrollment (1993–1998), 1 year, 3 years, 6 years). For analyses involving the WHI-CT random sample, the GLIMMIX procedure was applied with visit considered a random effect to examine the relationship of psychotropic medication use at WHI enrollment (1993–1998) with performance-based physical function (grip strength, chair stand, gait speed) cross-sectionally and cumulatively over time. Sensitivity analyses were conducted to examine patterns of psychotropic medication use between WHI enrollment (1993–1998) and 3-year follow-up visits in relation to change in self-reported physical function between WHI enrollment (1993–1998) and the last available follow-up visit. Furthermore, key analyses were repeated after stratifying by levels of depressive and insomnia symptoms. All multivariable models were adjusted for age, race, ethnicity, education, household income, marital status, smoking status, alcohol consumption, physical activity, BMI, cardiovascular disease, hypertension, hyperlipidemia, diabetes, symptoms of depression, and insomnia, as well as self-rated health. Complete subject analyses were performed, and two-tailed statistical tests were assessed at an alpha level of 0.05.

## Results

### Study flowchart

**Fig. 1:**
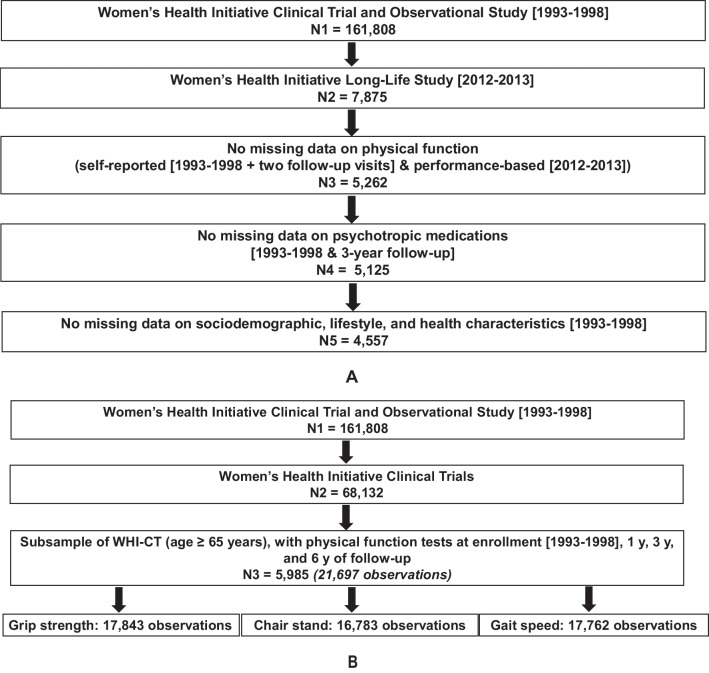
Study flowchart—Women’s Health Initiative Long Life Study and Women’s Health Initiative Clinical Trials (age ≥ 65 years) subsamples. Notes: *Women’s Health Initiative:* The enrollment visit for Women’s Health Initiative Clinical Trials and Observational Study occurred between 1993 and 1998. *Women’s Health Initiative Long Life Study* (Fig. 1A.): The final analytic sample consists of 4557 women who, in 2012–2013, participated in the Women’s Health Initiative Long Life Study, had no missing data on self-reported and performance-based physical function scores, psychotropic medication use at enrollment (1993–1998), and 3-year follow-up, as well as socio-demographic, lifestyle, and health characteristics at enrollment (1993–1998). Self-reported physical function was assessed at enrollment (1993–1998) and the last available follow-up visit, using the RAND-36 scale. The last available follow-up visit for self-reported physical function occurred an average of 22 (± 2.8) (range: 12–27) years after the enrollment (1993–1998) visit. Performance-based physical function was assessed at the 2012–2013 Women’s Health Initiative Long Life Study visit using the Short Physical Performance Battery. *Women’s Health Initiative Clinical Trials (age ≥ 65 years)* (Fig. 1B.): The final analytic sample consists of 5985 Women’s Health Initiative Clinical Trials participants (≥ 65 years of age at WHI enrollment) who had performance-based physical function tests (grip strength, chair stand, gait speed) at enrollment (1993–1998), 1-year, 3-year, and 6-year follow-up visits

Of 7875 2012–2013 WHI-LLS participants, 5262 had no missing data on self-reported or performance-based physical function measurements, and of those, 5125 had no missing data for medication use at WHI enrollment (1993–1998) and 3-year follow-up visits. The final analytic sample consisted of 4557 2012–2013 WHI-LLS (mean [±SD] age at WHI enrollment (1993–1998): 62.79 [±6.95] years) participants, whom, in addition to having complete physical function and medication use data, had no missing demographic, socioeconomic, lifestyle, and health data (Fig. 1a). An additional 5985 WHI-CT participants (≥ 65 years of age at WHI enrollment (1993–1998)) had complete performance-based physical function data available at WHI enrollment (1993–1998) and at 1-, 3-, and 6-year post-enrollment visits (a total of 21,697 observations) (Fig. 1b).

### Psychotropic medications among 2012–2013 WHI-LLS participants

The prevalence rates of antidepressant, anxiolytic, and sedative/hypnotic use at WHI enrollment (1993–1998) visit were 4.2%, 2.2%, and 2.4%, respectively. Antidepressant use differed among ethnic groups, and other variables measured at enrollment (1993–1998) that were directly associated with antidepressant use included lower physical activity scores, smoking, alcohol consumption, as well as history of cardiovascular disease, hypertension, and diabetes. Similarly, the use of anxiolytics was directly associated with history of cardiovascular disease, hypertension, and diabetes, and use of sedative/hypnotics was directly associated with BMI and history of diabetes measured at enrollment (1993–1998). Also, women with fair/poor self-rated health, high depressive, and/or insomnia symptom scores were more likely to be users of antidepressants, anxiolytic, and/or sedative/hypnotic medications at WHI enrollment (1993–1998) (Table 1).

**Table 1:** Associations of sociodemographic, lifestyle, and health characteristics at enrollment (1993–1998) with psychotropic medication use at enrollment (1993–1998) (*n* = 4557)—Women’s Health Initiative Long Life Study (2012–2013)

	Antidepressant		Anxiolytic		Sedative/hypnotic	
*Total:*	Yes	No	Yes	No	Yes	No
	189 (4.2%)	4368 (95.9%)	99 (2.2%)	4458 (97.8%)	111 (2.4%)	4446 (97.6%)
	***P*** **= 0.98**		***P*** **= 0.22**		***P*** **= 0.17**	
*WHI component:*						
CT	143 (4.2%)	3301 (95.9%)	80 (2.3%)	3364 (97.7%)	90 (2.6%)	3354 (97.4%)
OS	46 (4.2%)	1067 (95.9%)	19 (1.7%)	1094 (98.3%)	21 (1.9%)	1092 (98.1%)
	***P*** **= 0.24**		***P*** **= 0.68**		***P*** **= 0.24**	
*Age (years):*						
Continuous	62.2 ± 7.3	62.8 ± 6.9	62.5 ± 7.1	62.8 ± 6.9	63.6 ± 7.1	62.8 ± 6.9
	***P*** **= 0.12**		***P*** **= 0.59**		***P*** **= 0.39**	
0–54	79 (4.9%)	1512 (95.0%)	39 (2.5%)	1552 (97.6%)	39 (2.5%)	1552 (97.6%)
55–69	78 (3.6%)	2069 (96.4%)	42 (1.9%)	2105 (98.0%)	47 (2.2%)	2100 (97.8%)
70–79+	32 (3.9%)	787 (96.1%)	18 (2.2%)	801 (97.8%)	25 (3.0%)	794 (96.9%)
	***P*** **< 0.0001**		***P*** **= 0.88**		***P*** **= 0.21**	
*Race:*						
American Indian/Alaska Native	0 (0.0%)	7 (100.0%)	0 (0.0%)	7 (100.0%)	0 (0.0%)	7 (100.0%)
Black	39 (2.7%)	1409 (97.3%)	32 (2.2%)	1416 (97.8%)	24 (1.7%)	1424 (98.3%)
White	131 (4.6%)	2726 (95.4%)	61 (2.1%)	2796 (97.9%)	81 (2.8%)	2776 (97.2%)
More than one race	11 (13.3%)	72 (86.8%)	1 (1.2%)	82 (98.8%)	2 (2.4%)	81 (97.6%)
Unknown/not reported	8 (4.9%)	154 (95.1%)	5 (3.1%)	157 (96.9%)	158 (97.5%)	4 (2.5%)
	***P*** **= 0.55**		***P*** **= 0.84**		***P*** **= 0.95**	
*Ethnicity:*						
Not Hispanic/Latino	155 (4.1%)	3653 (95.9%)	82 (2.2%)	3726 (97.8%)	93 (2.4%)	3715 (97.6%)
Hispanic/Latino	34 (4.5%)	714 (95.5%)	17 (2.3%)	732 (97.7%)	18 (2.4%)	731 (97.6%)
	***P*** **= 0.51**		***P*** **= 0.36**		***P*** **= 0.21**	
*Marital status:*						
Married/partnered	116 (4.3%)	2602 (95.7%)	66 (2.4%)	2652 (97.6%)	64 (%)	2654 (%)
Single	6 (3.6%)	163 (96.4%)	4 (2.4%)	165 (97.6%)	8 (%)	161 (%)
Divorced	41 (4.7%)	838 (95.3%)	18 (2.0%)	861 (97.9%)	18 (%)	861 (%)
Widowed	26 (3.3%)	765 (96.7%)	11 (1.4%)	780 (98.6%)	21 (%)	770 (%)
	***P*** **= 0.68**		***P*** **= 0.18**		***P*** **= 0.45**	
*Education:*						
Less than high school	6 (3.2%)	182 (96.8%)	5 (2.7%)	183 (97.3%)	3 (1.6%)	185 (98.4%)
High school graduate	34 (4.7%)	697 (95.4%)	23 (3.2%)	708 (96.8%)	23 (3.2%)	708 (96.9%)
Some college	75 (4.4%)	1646 (95.6%)	37 (2.2%)	1684 (97.9%)	43 (2.5%)	1678 (97.5%)
College graduate	74 (3.9%)	96.1 (96.1%)	34 (1.8%)	1883 (98.2%)	42 (2.2%)	1875 (97.8%)
	***P*** **= 0.09**		***P*** **= 0.91**		***P*** **= 0.61**	
*Household income:*						
< $20,000	37 (5.6%)	620 (94.4%)	16 (2.4%)	641 (97.6%)	17 (2.6%)	640 (97.4%)
$20,000–$49,999	91 (4.4%)	1992 (95.6%)	48 (2.3%)	2035 (97.7%)	57 (2.7%)	2026 (97.3%)
$50,000–$99,999	46 (3.5%)	1254 (96.5%)	25 (1.9%)	1275 (98.1%)	26 (2.0%)	1274 (98.0%)
>$100,000	8 (2.4%)	333 (97.6%)	7 (2.0%)	334 (97.9%)	6 (1.8%)	335 (98.2%)
Unknown	7 (3.9%)	169 (96.0%)	3 (1.7%)	173 (98.3%)	5 (2.8%)	171 (97.2%)
	***P*** **= 0.002**		***P*** **= 0.58**		***P*** **= 0.05**	
*Smoking status:*						
Never	84 (3.3%)	2448 (96.7%)	51 (2.0%)	2481 (97.9%)	51 (2.0%)	2481 (97.9%)
Past	86 (4.9%)	1668 (95.1%)	43 (2.5%)	1711 (97.6%)	49 (2.8%)	1705 (97.2%)
Current	19 (7.0%)	252 (92.9%)	5 (1.9%)	266 (98.2%)	11 (4.1%)	260 (95.9%)
	***P*** **= 0.01**		***P*** **= 0.73**		***P*** **= 0.54**	
*Alcohol use:*						
Non-drinker	19 (3.8%)	483 (96.2%)	14 (2.8%)	488 (97.2%)	10 (1.9%)	492 (98.0%)
Former drinker	52 (5.9%)	819 (94.0%)	20 (2.3%)	851 (97.0%)	19 (2.2%)	852 (97.8%)
< 1 drink/week	68 (4.2%)	1558 (95.8%)	32 (1.9%)	1594 (98.0%)	37 (2.3%)	1589 (97.7%)
≥1 drink/week	50 (3.2%)	1508 (96.8%)	33 (2.1%)	1525 (97.9%)	45 (2.9%)	1513 (97.1%)
	***P*** **= 0.04**		***P*** **= 0.11**		***P*** **= 0.99**	
*Physical activity (Met-hours/week):*						
Continuous	10.3 ± 13.9	12.4 ± 13.8	10.5 ± 11.5	12.4 ± 13.9	12.4 ± 16.8	12.4 ± 13.7
	***P*** **= 0.001**		***P*** **= 0.73**		***P*** **= 0.02**	
*Body mass index (kg/m*^*2*^*):*						
Continuous	30.1 ± 6.6	28.7 ± 5.8	28.9 ± 7.1	28.8 ± 5.8	30.1 ± 6.7	28.7 ± 5.8
	***P*** **= 0.14**		***P*** **= 0.52**		***P*** **= 0.10**	
< 25	43 (3.4%)	1212 (96.6%)	27 (2.2%)	1228 (97.9%)	24 (1.9%)	1231 (98.1%)
25–29.9	68 (4.0%)	1638 (96.0%)	42 (2.5%)	1664 (97.6%)	38 (2.2%)	1668 (97.8%)
≥ 30	78 (4.9%)	1518 (95.1%)	30 (1.9%)	1566 (98.1%)	49 (3.1%)	1547 (96.9%)
*Medical history:*						
	***P*** **= 0.0002**		***P*** **= 0.006**		***P*** **= 0.80**	
*Cardiovascular disease:*						
Yes	49 (6.7%)	688 (93.4%)	26 (3.5%)	711 (96.5%)	17 (2.3%)	720 (97.7%)
No	140 (3.7%)	3680 (96.3%)	73 (1.9%)	3747 (98.1%)	94 (2.5%)	3726 (2.3%)
	***P*** **= 0.05**		***P*** **= 0.005**		***P*** **= 0.14**	
*Hypertension:*						
Yes	94 (4.8%)	1856 (95.2%)	56 (2.9%)	1894 (97.1%)	55 (2.8%)	1895 (97.2%)
No	95 (3.6%)	2512 (96.4%)	43 (1.7%)	2564 (98.4%)	56 (2.2%)	2551 (97.9%)
	***P*** **< 0.0001**		***P*** **= 0.03**		***P*** **= 0.04**	
*Diabetes:*						
Yes	56 (7.6%)	681 (92.4%)	24 (3.3%)	713 (96.7%)	26 (3.5%)	711 (96.5%)
No	133 (3.5%)	3687 (96.5%)	75 (1.9%)	3745 (98.0%)	85 (2.2%)	3735 (97.8%)
	***P*** **= 0.14**		***P*** **= 0.08**		***P*** **= 0.35**	
*Hyperlipidemia:*						
Yes	30 (5.3%)	537 (94.7%)	18 (3.2%)	549 (96.8%)	17 (3.0%)	550 (97.0%)
No	159 (3.9%)	3831 (5.3%)	81 (2.0%)	3909 (97.9%)	94 (2.4%)	3896 (97.6%)
	***P*** **< 0.0001**		***P*** **= 0.05**		***P*** **= 0.02**	
*Self-rated health:*						
Excellent/very good/good	164 (3.8%)	4135 (96.2%)	89 (2.1%)	4210 (97.9%)	99 (2.3%)	4200 (97.7%)
Fair/poor	25 (9.7%)	233 (90.3%)	10 (3.9%)	248 (96.1%)	12 (4.7%)	246 (95.4%)
	***P*** **< 0.0001**		***P*** **= 0.0006**		***P*** **= 0.005**	
*Depression score:*						
Continuous	2.6 ± 2.7	1.3 ± 1.9	2.2 ± 2.5	1.4 ± 1.9	1.9 ± 2.3	1.4 ± 1.9
	***P*** **< 0.0001**		***P*** **< 0.0001**		***P*** **< 0.0001**	
*Depression score:*						
> 0.06	133 (5.9%)	2095 (94.0%)	68 (3.05%)	2160 (96.9%)	69 (3.1%)	2159 (96.9%)
≤ 0.06	56 (2.4%)	2273 (97.6%)	31 (1.33%)	2298 (98.7%)	42 (1.8%)	2287 (98.2%)
	***P*** **< 0.0001**		***P*** **= 0.001**		***P*** **< 0.0001**	
*Insomnia score:*						
Continuous	8.0 ± 4.8	6.3 ± 4.4	7.8 ± 4.6	6.3 ± 4.4	9.8 ± 5.0	6.3 ± 4.4
	***P*** **= 0.0007**		***P*** **= 0.004**		***P*** **< 0.0001**	
*Insomnia score:*						
> 9	63 (5.9%)	990 (94.02)	35 (3.3%)	1018 (96.7%)	58 (5.5%)	995 (94.5%)
≤ 9	126 (3.6%)	3378 (96.4%)	64 (1.83%)	3440 (98.2%)	53 (1.5%)	3451 (98.5%)

Abbreviations: *WHI* Women’s Health Initiative

### Physical function among 2012–2013 WHI-LLS participants

Table 2 displays the associations of demographic, socioeconomic, lifestyle, and health characteristics at WHI enrollment (1993–1998) with physical function outcomes, namely, (a) self-reported physical function at enrollment (1993–1998) (mean ± SD: 84.05 ± 17.68, range: 0–100), (b) annualized change in self-reported physical function between the enrollment (1993–1998) visit and the last available follow-up visit (mean ± SD: −1.39 ± 1.44, range: −6.00–4.69), and (c) performance-based physical function at the 2012–2013 WHI-LLS follow-up visit (mean ± SD: 8.05 ± 2.69, range: 0–12). The mean (± SD) number of years elapsed between the enrollment (1993–1998) visit and the last available follow-up visit for assessing self-reported physical function was 21.99 (± 2.82), ranging between 12 and 27 years. Disparities in self-reported and performance-based physical function were observed according to most of the selected demographic, socioeconomic, lifestyle, and health characteristics.

**Table 2:** Associations of sociodemographic, lifestyle, and health characteristics at enrollment (1993–1998) with performance-based physical function at follow-up time (2012–2013), self-reported physical function at enrollment (1993–1998), and change in self-reported physical function between enrollment (1993–1998) and last available follow-up visit (*n* = 4557)—Women’s Health Initiative Long Life Study^a^

*Total:*	Performance-based physical function	Self-reported physical function	
	Follow-up visit (2012–2013)	Enrollment visit (1993–1998)	Change between enrollment and last follow-up visits^b^
	8.05 ± 2.69 (0–12)	84.05 ± 17.68 (0–100)	−1.39 ± 1.44 (−6.00–4.69)
	***P*** **< 0.0001**	***P*** **= 0.79**	***P*** **< 0.0001**
*WHI component:*			
CT	7.91 ± 2.73	84.01 ± 17.13	−1.53 ± 1.46
OS	8.48 ± 2.55	84.17 ± 19.29	−0.97 ± 1.31
*Age (years):*			
	***P*** **< 0.0001**	***P*** **< 0.0001**	***P*** **< 0.0001**
Continuous	*R* = −0.32	*R* = −0.11	*R* = −0.36
	***P*** **< 0.0001**	***P*** **< 0.0001**	***P*** **< 0.0001**
50–54	8.98 ± 2.26	86.26 ± 17.70	−0.80 ± 1.17
55–69	7.90 ± 2.63	83.73 ± 17.37	−1.53 ± 1.42
70–79+	6.64 ± 2.94	80.59 ± 17.86	−2.18 ± 1.53
	***P*** **= 0.009**	***P*** **= 0.0002**	***P*** **< 0.0001**
*Race:*			
American Indian/Alaska Native	8.71 ± 3.09	82.85 ± 23.77	−1.36 ± 1.57
Black	7.95 ± 2.57	82.27 ± 19.93	−1.05 ± 1.29
White	8.07 ± 2.77	84.86 ± 16.44	−1.60 ± 1.48
More than one race	7.78 ± 2.56	83.85 ± 15.64	−1.03 ± 1.33
Unknown/not reported	8.72 ± 2.34	85.89 ± 16.95	−0.91 ± 1.30
	***P*** **< 0.0001**	***P*** **= 0.0014**	***P*** **< 0.0001**
*Ethnicity:*			
Not Hispanic/Latino	7.89 ± 2.70	83.68 ± 17.86	−1.46 ± 1.45
Hispanic/Latino	8.86 ± 2.51	85.93 ± 16.58	−1.02 ± 1.36
	***P*** **< 0.0001**	***P*** **= 0.0002**	***P*** **< 0.0001**
*Marital status:*			
Married/partnered	8.22 ± 2.68	84.69 ± 16.87	−1.33 ± 1.43
Single	7.76 ± 2.82	81.83 ± 20.48	−1.28 ± 1.46
Divorced	8.17 ± 2.54	84.50 ± 18.22	−1.23 ± 1.41
Widowed	7.39 ± 2.80	81.83 ± 18.92	−1.79 ± 1.47
	***P*** **= 0.03**	***P*** **< 0.0001**	***P*** **= 0.18**
*Education:*			
Less than high school	7.74 ± 2.79	79.41 ± 20.91	−1.52 ± 1.52
High school graduate	7.88 ± 2.64	81.66 ± 19.09	−1.47 ± 1.50
Some college	8.04 ± 2.75	82.84 ± 18.460	−1.35 ± 1.46
College graduate	8.16 ± 2.66	86.50 ± 15.63	−1.39 ± 1.39
	***P*** **< 0.0001**	***P*** **< 0.0001**	***P*** **< 0.0001**
*Household income:*			
< $20,000	7.37 ± 2.75	77.57 ± 21.53	−1.45 ± 1.58
$20,000–$49,999	7.89 ± 2.72	83.87 ± 17.05	−1.50 ± 1.44
$50,000–$99,999	8.47 ± 2.58	86.88 ± 15.09	−1.29 ± 1.36
>$100,000	8.69 ± 2.53	88.98 ± 14.90	−1.10 ± 1.32
	***P*** **= 0.15**	***P*** **= 0.15**	***P*** **= 0.37**
*Smoking status:*			
Never	8.09 ± 2.73	84.50 ± 17.42	−1.39 ± 1.47
Past	8.03 ± 2.65	83.44 ± 17.82	−1.38 ± 1.42
Current	7.77 ± 2.63	83.76 ± 19.12	−1.51 ± 1.40
	***P*** **< 0.0001**	***P*** **< 0.0001**	***P*** **= 0.08**
*Alcohol use:*			
Non-drinker	7.88 ± 2.91	81.80 ± 20.14	−1.42 ± 1.53
Former drinker	7.55 ± 2.73	78.59 ± 21.11	−1.28 ± 1.48
< 1 drink/week	8.20 ± 2.62	84.65 ± 16.39	−1.39 ± 1.41
≥1 drink/week	8.24 ± 2.64	87.20 ± 15.03	−1.44 ± 1.42
	***P*** **< 0.0001**	***P*** **< 0.0001**	***P*** **= 0.29**
*Physical activity (Met-hours/week):*			
Continuous	*R* = 0.12	*R* = 0.24	*R* = −0.015
*Body mass index (kg/m*^*2*^*):*			
	***P*** **< 0.0001**	***P*** **< 0.0001**	***P*** **= 0.67**
Continuous	*R* = −0.21	*R* = −0.38	*R* = −0.006
	***P*** **< 0.0001**	***P*** **< 0.0001**	***P*** **= 0.06**
< 25	8.67 ± 2.54	90.62 ± 12.63	−1.35 ± 1.47
25–29.9	8.22 ± 2.58	86.51 ± 14.70	−1.36 ± 1.42
≥ 30	7.39 ± 2.79	76.27 ± 20.87	−1.46 ± 1.45
*Medical history:*			
	***P*** **< 0.0001**	***P*** **< 0.0001**	***P*** **= 0.02**
*Cardiovascular disease:*			
Yes	7.38 ± 2.83	76.66 ± 20.92	−1.50 ± 1.49
No	8.18 ± 2.65	85.47 ± 16.62	−1.37 ± 1.43
	***P*** **< 0.0001**	***P*** **< 0.0001**	***P*** **< 0.0001**
*Hypertension:*			
Yes	7.52 ± 2.78	80.66 ± 19.14	−1.56 ± 1.47
No	8.45 ± 2.55	86.58 ± 16.05	−1.26 ± 1.41
	***P*** **< 0.0001**	***P*** **< 0.0001**	***P*** **< 0.0001**
*Diabetes:*			
Yes	7.25 ± 2.91	77.78 ± 20.09	−1.60 ± 1.51
No	8.21 ± 2.63	85.26 ± 16.92	−1.35 ± 1.43
	***P*** **< 0.0001**	***P*** **< 0.0001**	***P*** **= 0.09**
*Hyperlipidemia:*			
Yes	7.55 ± 2.74	79.92 ± 19.09	−1.49 ± 1.54
No	8.12 ± 2.68	84.64 ± 17.39	−1.38 ± 1.43
	***P*** **< 0.0001**	***P*** **< 0.0001**	***P*** **< 0.0001**
*Self-rated health:*			
Excellent/very good/good	8.13 ± 2.67	85.36 ± 16.27	−1.41 ± 1.43
Fair/poor	6.76 ± 2.85	62.27 ± 24.69	−1.04 ± 1.58
	***P*** **= 0.0005**	***P*** **< 0.0001**	***P*** **= 0.0003**
*Depression score:*			
Continuous	*R* = −0.051	*R* = −0.14	*R* = 0.053
	***P*** **= 0.0002**	***P*** **< 0.0001**	***P*** **= 0.009**
*Depression score:*			
> 0.06	7.89 ± 2.71	81.86 ± 18.93	−1.33 ± 1.45
≤ 0.06	8.20 ± 2.68	86.15 ± 16.13	−1.44 ± 1.44
	***P*** **< 0.0001**	***P*** **< 0.0001**	***P*** **= 0.029**
*Insomnia score:*			
Continuous	*R* = −0.077	*R* = −0.18	*R* = −0.032
	***P*** **= 0.0008**	***P*** **< 0.0001**	***P*** **= 0.83**
*Insomnia score:*			
> 9	7.81 ± 2.79	79.35 ± 20.06	−1.40 ± 1.47
≤ 9	8.12 ± 2.66	85.46 ± 16.64	−1.38 ± 1.44

*Abbreviations: WHI* Women’s Health Initiative

^a^Performance-based physical function was assessed at the 2012–2013 Women’s Health Initiative Long Life Study visit using the Short Physical Performance Battery; Self-reported physical function was assessed at enrollment (1993–1998) and the last available follow-up visit, using the RAND-36 scale

^b^The last available follow-up visit for self-reported physical function occurred an average of 22 (±2.8) (range: 12–27) years after the 1993–1998 Women’s Health Initiative enrollment visit

### Psychotropic medications at enrollment in relation to self-reported physical function among 2012–2013 WHI-LLS participants

Table 3 presents the results of unadjusted and adjusted linear regression models for psychotropic medications at the 1993–1998 enrollment visit in relation to self-reported physical function at the enrollment (1993–1998) visit and to change in self-reported physical function between the enrollment (1993–1998) visit and the last available follow-up visit, 12 to 27 years later. We observed a negative cross-sectional relationship between antidepressant use and self-reported physical function at enrollment (1993–1998) which remained statistically significant after controlling for confounders (*β* = −6.27, 95% CI: −8.48, −4.07). Compared to non-users of any psychotropic medications, users of antidepressants alone had a significantly lower self-reported physical function at enrollment (1993–1998) (*β* = −6.65, 95% CI: −9.02, −4.28), after controlling for confounders. Antidepressant use was cross-sectionally related to lower self-reported physical function [< 78] (OR = 2.10, 95% CI: 1.48, 2.98) at enrollment (1993–1998). Also, the use of antidepressants alone (versus non-use of psychotropic medications) was cross-sectionally related to lower self-reported physical function [< 78] (OR = 2.18, 95% CI: 1.50, 3.18) at enrollment (1993–1998) (ESM 2, Table A.1). Unlike antidepressant medications, anxiolytics and sedative/hypnotics were not cross-sectionally related to self-reported physical function at enrollment (1993–1998). In fully adjusted regression models, combined patterns of psychotropic medication use at enrollment (1993–1998) were not significantly related to annualized change in self-reported physical function between enrollment (1993–1998) and the last available follow-up visits, a mean of 22 years later.

**Table 3:** Fixed-effects linear regression models for psychotropic medications at enrollment (1993–1998) as predictors of self-reported physical function at enrollment (1993–1998) and change in self-reported physical function between enrollment (1993–1998) and last available follow-up visits (*n* = 4557)—Women’s Health Initiative Long Life Study

	Baseline physical function		Change in physical function^b^	
	Unadjusted	Adjusted^a^	Unadjusted	Adjusted^a^
	*β* (95% CI)	*β* (95% CI)	*β* (95% CI)	*β* (95% CI)
Medication type:				
Antidepressant (yes vs. no)	−10.10 (−12.66, −7.55)	−6.27 (−8.48, −4.07)	0.066 (−0.14, 0.27)	0.078 (−0.12, 0.27)
Anxiolytic (yes vs. no)	−4.66 (−8.18, −1.14)	−2.15 (−5.15, 0.84)	−0.10 (−0.39, 0.18)	−0.12 (−0.38, 0.14)
Sedative/hypnotic (yes vs. no)	−4.84 (−8.17, −1.52)	−1.28 (−4.13, 1.56)	−0.33 (−0.61, −0.063)	−0.22 (−0.48, 0.027)
Patterns of use:				
None	Ref.	Ref.	Ref.	Ref.
Antidepressant only	−10.43 (−13.18, −7.68)	−6.64 (−9.03, −4.29)	0.11 (−0.11, 0.34)	0.12 (−0.085, 0.33)
Anxiolytic only	−4.52 (−8.53, −0.52)	−1.71 (−5.13, 1.71)	−0.12 (−0.45, 0.20)	−0.16 (−0.46, 0.14)
Sedative/hypnotic only	−4.77 (−8.37, −1.16)	−1.87 (−4.94, 1.23)	−0.23 (−0.53, 0.063)	−0.14 (−0.42, 0.13)
Combined	−8.33 (−14.25, −2.41)	−3.58 (−8.66, 1.49)	−0.32 (−0.81, 0.16)	−0.24 (−0.68, 0.21)

^a^Adjusted for age (continuous), race, ethnicity, education, household income, marital status, smoking status, alcohol consumption, physical activity, body mass index (categorical), cardiovascular disease, hypertension, hyperlipidemia, diabetes, depressive symptoms (categorical), insomnia symptoms (categorical), and self-rated health, as described in Tables 1 and 2

^b^Physical function score at the last available follow-up visit subtracted from physical function score at the enrollment visit divided by the duration of follow-up between the two visits. The last available follow-up visit for self-reported physical function occurred an average of 22 (± 2.8) (range: 12–27) years after the 1993–1998 Women’s Health Initiative enrollment visit

### Psychotropic medications between enrollment and 3-year follow-up in relation to self-reported physical function among 2012–2013 WHI-LLS participants

Sensitivity analyses with repeated assessments of psychotropic medications at enrollment (1993–1998) and then again at 3-year follow-up indicated that women who reported sedative/hypnotic use at enrollment (1993–1998) but not at the 3-year follow-up visit experienced a decline in self-reported physical function between enrollment (1993–1998) and the last available follow-up visit (*β* = −0.36, 95% CI: −0.65, −0.079) in a fully adjusted regression model (ESM 2, Table A.2).

### Psychotropic medications in relation to performance-based physical function among 2012–2013 WHI-LLS participants

Table 4 presents unadjusted and adjusted linear regression models for the use of psychotropic medications at enrollment (1993–1998) in relation to performance-based physical function at the 2012–2013 WHI-LLS follow-up visit. Unadjusted models suggested a negative relationship between antidepressants taken alone (or in combination with sedative/hypnotics) at enrollment (1993–1998) and performance-based physical function at the 2012–2013 WHI-LLS follow-up visit; however, these relationships became statistically non-significant after controlling for confounders. By contrast, in a multivariable logistic regression model, antidepressant use at enrollment (1993–1998) was associated with 50% increased odds of lower (< 10) performance-based physical function at the 2012–2013 WHI-LLS visit (OR = 1.53, 95% CI: 1.05, 2.21) (ESM 2, Table A.3).

**Table 4:** Fixed-effect linear regression models for psychotropic medications at enrollment (1993–1998) as predictors of performance-based physical function at the Women’s Health Initiative Long Life Study follow-up visit (2012–2013) (*n* = 4557)—Women’s Health Initiative Long Life Study^a^

	Unadjusted	Adjusted^b^
	*β* (95% CI)	*β* (95% CI)
Medication type:		
Antidepressant (yes vs. no)	−0.62 (−1.01, −0.23)	−0.35 (−0.71, 0.006)
Anxiolytic (yes vs. no)	−0.24 (−0.78, 0.29)	−0.063 (−0.54, 0.42)
Sedative / Hypnotic (yes vs. no)	−0.45 (−0.96, 0.055)	−0.11 (−0.56, 0.35)
Patterns of use:		
None	Ref.	Ref.
Antidepressant only	−0.57 (−0.99, −0.15)	−0.29 (−0.68, 0.08)
Anxiolytic only	−0.26 (−0.88, 0.35)	−0.058 (−0.61, 0.49)
Sedative/hypnotic only	−0.29 (−0.85, 0.26)	−0.020 (−0.52, 0.48)
Combined	−0.86 (−1.76, 0.054)	−0.46 (−1.28, 0.36)

^a^Performance-based physical function was assessed at the 2012–2013 Women’s Health Initiative Long Life Study visit using the Short Physical Performance Battery

^b^Adjusted for age (continuous), race, ethnicity, education, household income, marital status, smoking status, alcohol consumption, physical activity, body mass index (categorical), cardiovascular disease, hypertension, hyperlipidemia, diabetes, depressive symptoms (categorical), insomnia symptoms (categorical), and self-rated health, as described in Tables 1 and 2

### Psychotropic medications in relation to performance-based physical function measures among WHI-CT participants

Table 5 presents mixed-effects linear regression models for use of antidepressant, anxiolytic, and sedative/hypnotic medications at WHI enrollment (1993–1998) in relation to repeated measures (enrollment (1993–1998), 1-year, 3-year, 6-year post-enrollment) of grip strength (17,843 observations), chair stands (16,783 observations), and gait speed (17,762 observations) among a random sample of 21,325 observations linked to 5985 WHI-CT participants, ≥ 65 years of age at enrollment (1993–1998). In fully adjusted models, use of antidepressants was cross-sectionally related to marginally worse performance on grip strength (users vs. non-users: 21.46 (1.92) vs. 21.76 (1.89); *P* = 0.0071), chair stand (users vs. non-users: 6.48 (0.16) vs. 6.71 (0.12); *P* < 0.0001), and gait speed (users vs. non-users: 7.06 (0.39) vs. 6.82 (0.30); *P* = 0.039) tests at enrollment (1993–1998). Use of anxiolytics was cross-sectionally related to marginally worse performance only on the chair stand test (users vs. non-users: 6.54 (0.18) vs. 6.71 (0.12); *P* = 0.0011) at enrollment (1993–1998). By contrast, hypnotics/sedatives were not cross-sectionally related to performance-based physical function measures at enrollment (1993–1998). Use of psychotropic medications at enrollment (1993–1998) was not associated with a decline in any of the performance-based physical function measures.

**Table 5:** Mixed-effect linear regression models for psychotropic medications at enrollment (1993–1998) as predictors of performance-based physical function measurements (grip strength, chair stands, gait speed) at enrollment (1993–1998) [Medication] and cumulatively over time (enrollment [1993–1998], 1-year, 3-year, and 6-year follow-up visits) [Medication x Visit]—Women’s Health Initiative Clinical Trials (≥ 65 years at enrollment) (*n* = 5985)

	Unadjusted		Adjusted^a^	
	*F*	*P value*	*F*	*P value*
	**Grip strength (*n*** **= 17,843 observations)**			
Antidepressant (yes vs. no):				
Medication	13.51	0.0002	7.25	0.0071
Medication × visit	0.21	0.89	0.21	0.89
Anxiolytic (yes vs. no):				
Medication	3.09	0.079	0.09	0.77
Medication × visit	0.39	0.76	0.39	0.75
Sedative/hypnotic (yes vs. no):				
Medication	0.92	0.33	0.49	0.48
Medication × visit	0.26	0.85	0.33	0.80
	**Chair stand (*n*** **= 16,783 observations)**			
Antidepressant (yes vs. no):				
Medication	78.26	< 0.0001	32.40	< 0.0001
Medication × visit	0.44	0.72	0.83	0.48
Anxiolytic (yes vs. no):				
Medication	52.46	< 0.0001	10.57	0.0011
Medication × visit	0.42	0.73	0.63	0.59
Sedative/hypnotic (yes vs. no):				
Medication	11.53	0.0007	1.48	0.22
Medication x visit	1.04	0.37	1.23	0.29
	**Gait speed (*n*** **= 17,762 observations)**			
Antidepressant (yes vs. no):				
Medication	8.92	0.0028	4.24	0.039
Medication × visit	0.19	0.90	0.24	0.87
Anxiolytic (yes vs. no):				
Medication	1.86	0.17	0.00	0.98
Medication × visit	1.60	0.18	1.53	0.20
Sedative/hypnotic (yes vs. no):				
Medication	0.42	0.52	0.00	0.98
Medication × visit	0.52	0.66	0.55	0.64

^a^Adjusted for age (continuous), race, ethnicity, education, household income, marital status, smoking status, alcohol consumption, physical activity, body mass index (categorical), cardiovascular disease, hypertension, hyperlipidemia, diabetes, depressive symptoms (categorical), insomnia symptoms (categorical), and self-rated health, as described in Tables 1 and 2

### Key analyses stratified by depressive and insomnia symptoms

Multiple linear regression models for relationships of psychotropic medication (*i.e.*, antidepressant and sedative/hypnotic) use at enrollment (1993–1998) with key continuous physical function outcomes are displayed after stratifying by levels of depressive (Table A.4.) and insomnia (Table A.5.) symptoms at enrollment (1993–1998). At enrollment (1993–1998), use of hypnotics was cross-sectionally associated with grip strength among women with low (≤ 9) and high (> 9) levels of insomnia symptoms, with a significant interaction effect (*P*_interaction_ = 0.006). Among women with WHIIRS score ≤ 9, grip strength was significantly lower among users (21.72 [0.70]) vs. non-users (22.13 [0.44]) of sedative/hypnotics (*P* = 0.04), at enrollment (1993–1998). Conversely, among women with WHIIRS score > 9, grip strength was significantly higher among users (22.23 [0.88]) vs. non-users (21.26 [0.68]) of sedative/hypnotics (*P* = 0.04), at enrollment (1993–1998). There were no other significant interactions between antidepressant use and depressive symptoms or between hypnotic use and insomnia symptoms in relation to the key physical function outcomes.

## Discussion

In this epidemiological study, we analyzed data on a sub-sample of > 4000 WHI participants who, in 2012–2013, participated in the WHI-LLS and found that use of anxiolytics was not cross-sectionally or longitudinally associated with self-reported or performance-based physical function, whereas antidepressant use assessed at WHI enrollment (1993–1998) was cross-sectionally (but not longitudinally) related to worse self-reported physical function and was associated with worse performance-based physical function at the 2012–2013 WHI-LLS follow-up visit. Unexpected results of analyses involving this sub-sample of > 4000 WHI participants were that, compared to non-users, those using sedative/hypnotics at enrollment (1993–1998) but not at the 3-year follow-up visit self-reported a decline in physical function over an average of 22 years of follow-up. Mixed models constructed using a subsample of > 5000 WHI-CT participants (≥ 65 years of age at enrollment (1993–1998)) suggested that (a) psychotropic medication use at enrollment (1993–1998) was not longitudinally related to performance-based physical function tests, (b) sedative/hypnotic use at enrollment (1993–1998) was not cross-sectionally related to performance-based physical function tests, but the relationship with grip strength was dependent on level of insomnia symptoms; (c) use of antidepressants or anxiolytics at enrollment (1993–1998) was cross-sectionally, but marginally, related to worse performance on the chair stand test; and, (d) antidepressant use at enrollment (1993–1998) was cross-sectionally, but marginally, related to worse performance on grip strength and gait speed tests.

A thorough review of the literature indicated that evidence linking psychotropic medication use to self-reported and performance-based physical function remains scarce. However, there are numerous studies that have linked physical function to underlying conditions for which the psychotropic medications we studied are often prescribed. It is important to note that, in clinical practice, symptoms and diagnoses may not match with, and therefore, are not acceptable *proxies* for medication type. Despite having symptoms and/or being diagnosed with a specific condition, individuals may not be treated with the types of psychotropic medications under study. Nevertheless, published studies examining the association of depression and/or anxiety with physical function may provide insight into our broadly defined study question. For instance, a community-based prospective cohort study of individuals, > 65 years of age, suggested that late-life depression may be associated with a greater risk of mortality with physical inactivity and hand-grip strength partly mediating this association [7]. Another cohort study of community-dwelling participants, 80 years and older, found that depression was associated with lower 4-m walking test and SPPB scores and increased number of impaired Instrumental Activities of Daily Living, though not significantly associated with hand-grip strength or Basic Activities of Daily Living scores [8]. Analyses of 5-year longitudinal cohort data on adults, 70–79 years at baseline, from the Health, Aging and Body Composition (Health ABC) study suggested that anxiety symptoms were not associated with declines in objectively measured physical performance but were associated with declines in self-reported functioning over a 5-year follow-up period [77]. An evaluation of baseline psychiatric status in relation to a 6-year trajectory of objective physical function in the Netherlands Study of Depression and Anxiety [78] suggested no differences in the rate of decline over time, whereas women with current depression/anxiety had poorer hand-grip strength compared to non-anxious/depressed women during the 6-year follow-up [78]. Finally, mixed growth curve models using annual surveys of community-dwelling adults with chronic conditions suggested significant concurrent and longitudinal associations of anxiety with self-reported physical function, cognitive function, and satisfaction with social roles, independent of depression severity [5]. Whereas both anxiety and depression exhibited similar effect sizes in their unique relationships with each outcome, depression was more strongly associated with satisfaction with social roles and physical function than anxiety [5].

Compared to studies of depression and anxiety, there has been more interest in cross-sectional and longitudinal evaluations of the relationship between sleep parameters and physical function [15, 17–19, 21–27]. Depression, anxiety, and sleep disorders are highly enmeshed with no clear causal relationship among them. Psychotropic medications prescribed for these indications only add to the difficulty in understanding how variations in physical function found in our analyses relate to each of the underlying conditions. Although use of sedative/hypnotics was not cross-sectionally related to physical function in this study, the finding that inconsistent use of sedative/hypnotics may be a risk factor for decline in physical function is unexpected and novel, although it might reflect a propensity for medication adherence or a beneficial effect of sedative/hypnotics on sleep quality, and, consequently, on physical function. Study replication is needed to confirm these preliminary findings.

Previous studies have suggested that psychotropic medications may increase morbidity and mortality risks and have a negative impact on physical function among older adults. For example, An *et al.* examined the relationship between antidepressant use and onset of functional limitations pertaining to physical mobility, large muscle function, activities of daily living, gross motor function, fine motor function, and instrumental activities of daily living, among Health and Retirement Study participants, showing that antidepressant use for ≥ 12 months was associated with greater risk of functional limitation by 8%, whereas the relationship between use of antidepressants for < 12 months and functional limitation was statistically non-significant [29]. Antidepressant use was associated with a greater risk of functional limitation by 8% among currently non-depressed participants but not currently depressed participants [29], highlighting the complex entanglement of psychotropic medications with underlying conditions and indications. Blazer *et al.* analyzed sociodemographic and health characteristics in relation to anxiolytic, sedative, and hypnotic medication in a community-based, bi-racial cohort of 4000 older adults followed for 10 years [12]. Overall, 13.3% of subjects were taking these medications at baseline, with the proportion 10 years later only modestly less at 11.8% [12]. Correlates of use at baseline and follow-up were female gender, white race, depression symptoms, poor self-rated health, health services use, and physical disability defined based on items from the modified Katz Activities of Daily Living scale, the Instrumental Activities of Daily Living scale, and the Rosow-Breslow Physical Health Scale [12]. Finally, Hartz and Ross analyzed data on 148,938 postmenopausal women, aged 50–79 years at baseline, from the WHI to evaluate baseline hypnotic use in relation to risks of mortality, myocardial infarction, stroke, diabetes, and seven types of cancer, over a median follow-up time of 8 years [32], yielding results that are somewhat inconsistent with our study. For women who used hypnotic medications almost daily, the age-adjusted hazard ratio (HR) for mortality was 1.62 [32]. Greater hypnotic use was associated with less healthy levels of self-reported physical function, general health, and smoking at baseline [32]. After adjustment for these factors, the HR for almost daily hypnotic use was 1.14 for mortality and 1.53 for melanoma but not significantly associated with increased incidence of other diseases or disorders examined [32]. Less frequent hypnotic use and most types of sleeping difficulties were not associated with mortality, but sleeping more than 10 h a night had a risk-adjusted HR for mortality of 1.28 [32].

A strength of the study is that the WHI study involves detailed data collection at enrollment which facilitates the evaluation of hypothesized relationships taking key confounders into consideration. Second, analyses of WHI data could be generalized to postmenopausal women of diverse racial/ethnic backgrounds that reside in various geographical areas within the U.S. However, there are several limitations necessitating cautious interpretation of study findings. First, data from WHI clinical trials and observational studies were combined in these analyses, although they consist of multiple studies that differ in terms of design and eligibility criteria. Second, missing data on exposure, outcome, and covariate variables may have resulted in selection bias. Third, given that most of the variables were assessed through self-report, non-differential misclassification may have resulted in the underestimation of exposure-outcome relationships. Fourth, residual confounding, due to unmeasured or inadequately measured confounders as well as confounding by indication, remains a concern for observational study designs. For instance, the relationship between psychotropic medication use and physical function may be confounded by complex characteristics such as access to and use of healthcare services, which were not adequately evaluated in this study. Although multivariable models controlled for depressive and insomnia symptoms, we were unable to control for anxiety symptoms and could not ascertain the specific indications for the use of psychotropic medications. Thus, disentangling the role of a psychotropic medication from that of its indicated health condition remains an issue to be addressed in future studies. Fifth, it is difficult to ascertain whether a change in the use of psychotropic medications had occurred in the intervening period between baseline and follow-up visits. Accordingly, future studies should incorporate repeated measurements of psychotropic medication use and physical function, which would also elucidate the potential for reverse causality. It is worth noting that a causal relationship between psychotropic medication use and physical function can only be definitively established in the context of an experimental design. Sixth, despite the relatively large sample size, these analyses were adequately powered for evaluating the relationship of antidepressant use with physical function, but likely underpowered to detect marginal differences between users and non-users of other psychotropic medications, especially when less than 5% of the study sample reported using anxiolytics and sedative/hypnotics. A recently published Data Brief by the National Center for Health Statistics estimated the 12-month prevalence rates of any mental health treatment, prescription medication, and counseling or therapy from a mental health professional, at 20.3%, 16.5%, and 10.1%, respectively, in 2020 [79]. Thus, study results may not generalize to contemporaneous populations of postmenopausal women, whereby use of psychotropic medications has become more commonplace in recent years. Finally, the WHI is not population-based but involves volunteers at clinical centers, specifically targeting postmenopausal women. Therefore, its generalizability to men as well as younger and less-educated women is not possible.

In conclusion, the use of antidepressants, not anxiolytics or hypnotics, was cross-sectionally related to worse self-reported physical function and was also associated with worse performance-based physical function > 20 years later, among postmenopausal women. These preliminary findings should be validated using larger samples with repeated measurements of psychotropic medications and physical function to determine the bi-directional relationship between these two constructs. Complex relationships involving hypnotic/sedatives necessitate further investigation. Future studies should explore the use of advanced analytic techniques such as Mendelian randomization or negative controls to address the issue of confounding by indication. Reliance on large claims and electronic health record databases can aid in answering questions pertaining to the cost-effectiveness of psychotropic medication use among different patient subgroups defined according to their disease severity and duration.

## Supplementary information

Supplementary file 1 (DOCX 52 kb)

Supplementary file 2 (DOCX 35 kb)

## Data Availability

Data can be made available from the corresponding author upon reasonable request.

## Declarations

### Disclaimer

The views expressed in this article are those of the authors and do not reflect the official policy of the A.T. Augusta Military Medical Center, the Defense Health Agency, Department of Defense, or the U.S. Government. Any discussion or mention of commercial products or brand names does not imply or support any endorsement by the Federal Government.
