# Supplementary material for: Relationship of psychotropic medication use with physical function among postmenopausal women
Source: GeroScience. 2024 Mar 22;46(6):5797–817. doi: 10.1007/s11357-024-01141-z (PMC11493997; doi:10.1007/s11357-024-01141-z)
Supplement: Supplementary file 1 — (DOCX 52 kb) [file 11357_2024_1141_MOESM1_ESM.docx]

**ESM 1 – Appendix Methods:**

Relationship of psychotropic medication use with physical function among postmenopausal women

Hind A. Beydoun, PhD, MPH ^a, b*^; May A. Beydoun, PhD, MPH ^b^; Edward Kwon, MD ^c^; Brook T. Alemu ^d^; Alan B. Zonderman, PhD ^b^; Robert Brunner, PhD ^e^

*^a^ Department of Research Programs, A.T. Augusta Military Medical Center, Fort Belvoir, VA, USA*

*^b^ Laboratory of Epidemiology and Population Sciences, National Institute on Aging, NIA/NIH/IRP, Baltimore, MD, USA*

*^c^ Department of Family Medicine, A.T. Augusta Military Medical Center, Fort Belvoir, VA, USA*

*^d^ Health Sciences Program, School of Health Sciences, Western Carolina University, Cullowhee, NC, USA*

*^e^ Department of Family and Community Medicine (Emeritus), School of Medicine, University of Nevada (Reno), NV, USA*

**Corresponding author’s email address:** [hind.baydoun@nih.gov](mailto:hind.baydoun@nih.gov)

**Psychotropic medications:** Use of *antidepressant* (α-2 receptor antagonists (Tetracyclics) [580300], monoamine oxidase (MAO) inhibitors [581000], modified cyclics [581200], selective serotonin reuptake inhibitors [581600], serotonin-norepinephrine reuptake inhibitors [581800], tricyclic agents [582000], miscellaneous antidepressants [583000], antidepressant combinations [589900, 589980, 589985, 589987, 589990]), *anxiolytic* (benzodiazepines [571000], benzodiazepine antagonists [571020], miscellaneous anti-anxiety agents [572000], antianxiety agent combinations [579900, 579990]), and *sedative/hypnotic* (barbiturate hypnotics [601000], non-barbiturate hypnotics [602000], benzodiazepine hypnotics [602010], non-benzodiazepine – GABA receptor modulators [602040], selective α-2-adrenoreceptor agonist sedatives [602060], selective melatonin receptor agonists [602500], antihistamine hypnotics [603000], antihistamine hypnotic combinations [603099], hypnotic combinations [609900, 609980, 609985]) medications among study participants were defined using the specified therapeutic class codes. The use of specific types of psychotropic medications at enrollment (1993-1998) among 161,808 participants from WHI-CTs and WHI-OS as well as 4,557 participants in the study sample is described below.

| **Psychotropic medications:** | **N=161,808** | **N=4,557** |
| --- | --- | --- |
| *Antidepressants:* | % | % |
| α-2 receptor antagonists (Tetracyclics) [580300] | 0.02 | 0.02 |
| Monoamine oxidase (MAO) inhibitors [581000] | 0.02 | 0.00 |
| Modified cyclics [581200] | 0.58 | 0.31 |
| Selective serotonin reuptake inhibitors [581600] | 3.40 | 2.02 |
| Serotonin-norepinephrine reuptake inhibitors [581800] | 0.00 | 0.00 |
| Tricyclic agents [582000] | 2.67 | 1.82 |
| Miscellaneous antidepressants [583000] | 0.51 | 0.24 |
| Antidepressant combinations  [589900, 589980, 589985, 589987, 589990] | 0.00 | 0.00 |
| *Anxiolytics:* | % | % |
| Benzodiazepines [571000] | 2.27 | 1.65 |
| Benzodiazepine antagonists [571020] | 0.00 | 0.00 |
| Miscellaneous anti-anxiety agents [572000] | 0.67 | 0.55 |
| Antianxiety agent combinations [579900, 579990] | 0.00 | 0.00 |
| *Sedative/hypnotics:* | % | % |
| Barbiturate hypnotics [601000] | 0.08 | 0.11 |
| Non-barbiturate hypnotics [602000] | 0.01 | 0.00 |
| Benzodiazepine hypnotics [602010] | 0.46 | 0.33 |
| Non-benzodiazepine – GABA receptor modulators [602040] | 0.29 | 0.22 |
| Selective α-2-adrenoreceptor agonist sedatives [602060] | 0.00 | 0.00 |
| Selective melatonin receptor agonists [602500] | 0.00 | 0.00 |
| Antihistamine hypnotics [603000] | 0.21 | 0.13 |
| Antihistamine hypnotic combinations [603099] | 1.69 | 1.69 |
| Hypnotic combinations [609900, 609980, 609985] | 0.00 | 0.00 |

For the 4,557 participants in the study sample, sample sizes for exposure variables at enrollment and change in exposure between enrollment and 3-year follow-up in relation to outcome variables at follow-up visits are shown below:

|  | **Total** | **Self-reported physical function** | | **Performance-based physical function** | |
| --- | --- | --- | --- | --- | --- |
|  |  | < 78 | ≥ 78 | < 10 | ≥ 10 |
| **Total** | 4557 | 1276 | 3281 | 3018 | 1539 |
| **Antidepressant use:** |  |  |  |  |  |
| Yes | 189 | 98 | 91 | 143 | 46 |
| No | 4368 | 1178 | 3190 | 2875 | 1493 |
| **Anxiolytic use:** |  |  |  |  |  |
| Yes | 99 | 41 | 58 | 69 | 30 |
| No | 4458 | 1235 | 3223 | 2949 | 1509 |
| **Sedative/Hypnotic use:** |  |  |  |  |  |
| Yes | 111 | 44 | 67 | 75 | 36 |
| No | 4446 | 1232 | 3214 | 2943 | 1503 |
| **Patterns of use at enrollment:** | | | | | |
| None | 4193 | 1112 | 3081 | 2759 | 1434 |
| Antidepressant only | 162 | 83 | 79 | 121 | 41 |
| Anxiolytic only | 75 | 29 | 46 | 51 | 24 |
| Sedative/Hypnotic only | 93 | 59 | 34 | 60 | 33 |
| Combined | 34 | 18 | 16 | 27 | 7 |
| **Patterns of use between enrollment and 3-year follow-up:** | | | | | |
| **Antidepressant use:** |  |  |  |  |  |
| None | 4271 | 1143 | 3128 | 2811 | 1460 |
| At enrollment only | 94 | 50 | 44 | 68 | 26 |
| At 3-year follow-up only | 97 | 35 | 62 | 64 | 33 |
| At enrollment and 3-year follow-up | 95 | 48 | 47 | 75 | 20 |
| **Anxiolytic use:** |  |  |  |  |  |
| None | 4427 | 1223 | 3204 | 2926 | 1501 |
| At enrollment only | 72 | 28 | 44 | 48 | 24 |
| At 3-year follow-up only | 31 | 12 | 19 | 23 | 8 |
| At enrollment and 3-year follow-up | 27 | 13 | 14 | 21 | 6 |
| **Sedative use:** |  |  |  |  |  |
| None | 4400 | 1213 | 3187 | 2906 | 1494 |
| At enrollment only | 85 | 34 | 51 | 52 | 33 |
| At 3-year follow-up only | 46 | 19 | 27 | 37 | 9 |
| At enrollment and 3-year follow-up | 26 | 10 | 16 | 23 | 3 |
