# Supplementary material for: Relationship of psychotropic medication use with physical function among postmenopausal women
Source: GeroScience. 2024 Mar 22;46(6):5797–817. doi: 10.1007/s11357-024-01141-z (PMC11493997; doi:10.1007/s11357-024-01141-z)
Supplement: Supplementary file 2 — (DOCX 35 kb) [file 11357_2024_1141_MOESM2_ESM.docx]

**ESM 2 – APPENDIX TABLES**

Relationship of psychotropic medication use with physical function among postmenopausal women

Hind A. Beydoun, PhD, MPH ^a, b*^; May A. Beydoun, PhD, MPH ^b^; Edward Kwon, MD ^c^; Brook T. Alemu ^d^; Alan B. Zonderman, PhD ^b^; Robert Brunner, PhD ^e^

*^a^ Department of Research Programs, A.T. Augusta Military Medical Center, Fort Belvoir, VA, USA*

*^b^ Laboratory of Epidemiology and Population Sciences, National Institute on Aging, NIA/NIH/IRP, Baltimore, MD, USA*

*^c^ Department of Family Medicine, A.T. Augusta Military Medical Center, Fort Belvoir, VA, USA*

*^d^ Health Sciences Program, School of Health Sciences, Western Carolina University, Cullowhee, NC, USA*

*^e^ Department of Family and Community Medicine (Emeritus), School of Medicine, University of Nevada (Reno), NV, USA*

**Corresponding author’s email address:** [hind.baydoun@nih.gov](mailto:hind.baydoun@nih.gov)

**Table A.1.** Psychotropic medications at enrollment (1993-1998) as predictors of low score (< 78 vs. ≥ 78) on self-reported physical function at enrollment (1993-1998) (n=4,557) – Women’s Health Initiative Long Life Study ^a^

|  | **Enrollment** | |
| --- | --- | --- |
|  | **Unadjusted** | **Adjusted ^b^** |
|  | **OR (95% CI)** | **OR (95% CI)** |
| **MEDICATION TYPE:** |  |  |
| Antidepressant (Yes vs. No) | 2.65 (1.97, 3.56) | 2.10 (1.48, 2.98) |
| Anxiolytic (Yes vs. No) | 1.72 (1.13, 2.60) | 1.47 (0.92, 2.38) |
| Sedative / Hypnotic (Yes vs. No) | 1.92 (1.30, 2.84) | 1.40 (0.88, 2.23) |
| **PATTERNS OF USE (DEFINITION 1):** |  |  |
| None | Ref. | Ref. |
| Antidepressant only | 2.66 (1.94, 3.66) | 2.18 (1.50, 3.18) |
| Anxiolytic only | 1.67 (1.03, 2.71) | 1.39 (0.79, 2.46) |
| Sedative / Hypnotic only | 1.75 (1.13, 2.69) | 1.38 (0.83, 2.29) |
| Antidepressant + Anxiolytic | 1.99 (0.73, 5.51) | 1.74 (0.57, 5.36) |
| Antidepressant + Sedative / Hypnotic | 4.99 (1.41, 17.75) | 1.47 (0.35, 6.24) |
| Anxiolytic + Sedative / Hypnotic | 2.49 (0.56, 11.18) | 1.74 (0.36, 8.39) |
| Antidepressant + Anxiolytic + Sedative / Hypnotic | -- | -- |
| **PATTERNS OF USE (DEFINITION 2):** |  |  |
| None | Ref. | Ref. |
| Antidepressant only | 2.66 (1.94, 3.66) | 2.18 (1.50, 3.18) |
| Anxiolytic only | 1.67 (1.03, 2.71) | 1.39 (0.79, 2.46) |
| Sedative / Hypnotic only | 1.75 (1.13, 2.69) | 1.38 (0.83, 2.29) |
| Combined | 2.96 (1.50, 5.83) | 1.87 (0.87, 4.06) |

^a^ Self-reported physical function was assessed using the RAND-36 scale; ^b^ Adjusted for age (continuous), race, ethnicity, education, household income, marital status, smoking status, alcohol consumption, physical activity, body mass index (categorical), cardiovascular disease, hypertension, hyperlipidemia, diabetes, depressive symptoms (categorical), insomnia symptoms (categorical), and self-rated health, as described in tables 1 and 2.

**Table A.2.** Patterns of psychotropic medications between enrollment (1993-1998) and 3-year follow-up visits as predictors of change in self-reported physical function between enrollment (1993-1998) and latest available follow-up visits (n=4,557) – Women’s Health Initiative Long Life Study

|  | **Change in physical function ^a^** | |
| --- | --- | --- |
|  | **β (95% CI)** | |
| **Model IIA: Antidepressant** | **Unadjusted** | **Adjusted ^b^** |
| None | Ref. | Ref. |
| At enrollment only | 0.19 (-0.10, 0.48) | 0.17 (-0.097, 0.44) |
| At 3-year follow-up only | 0.092 (-0.19, 0.38) | -0.066 (-0.33, 0.20) |
| At enrollment and 3-year follow-up | -0.05 (-0.34, 0.24) | -0.023 (-0.29, 0.25) |
| **Model IIB: Anxiolytic** |  |  |
| None | Ref. | Ref. |
| At enrollment only | -0.071 (-0.41, 0.26) | -0.086 (-0.39, 0.22) |
| At 3-year follow-up only | -0.12 (-0.63, 0.39) | -0.35 (-0.82, 0.12) |
| At enrollment and 3-year follow-up | -0.20 (-0.75, 0.35) | -0.086 (-0.39, 0.22) |
| **Model IIC: Sedative / Hypnotic** |  |  |
| None | Ref. | Ref. |
| At enrollment only | -0.44 (-0.75, -0.13) | -0.36 (-0.65, -0.079) |
| At 3-year follow-up only | -0.06 (-0.48, 0.35) | -0.16 (-0.55, 0.22) |
| At enrollment and 3-year follow-up | 0.011 (-0.55, 0.57) | 0.23 (-0.28, 0.74) |

^a^ Self-reported physical function was assessed using the RAND-36 scale; Self-reported physical function score at the last available follow-up visit was subtracted from self-reported physical function score at the enrollment visit and divided by the duration of follow-up between the two visits; The last available follow-up visit for self-reported physical function occurred an average of 22 (± 2.8) (range:12-27) years after the 1993-1998 Women’s Health Initiative enrollment visit. ^b^ Adjusted for age (continuous), race, ethnicity, education, household income, marital status, smoking status, alcohol consumption, physical activity, body mass index (categorical), cardiovascular disease, hypertension, hyperlipidemia, diabetes, depressive symptoms (categorical), insomnia symptoms (categorical), and self-rated health, as described in tables 1 and 2. *Abbreviations:* β = slope; CI = confidence interval.

**Table A.3.** Psychotropic medications at enrollment (1993-1998) as predictors of low score (< 10 vs. ≥ 10) on performance-based physical function at the WHI-LLS (2012-2013) visit (n=4,557) – Women’s Health Initiative Long Life Study ^a^

|  | **Unadjusted** | **Adjusted ^b^** |
| --- | --- | --- |
|  | **OR (95% CI)** | **OR (95% CI)** |
| **MEDICATION TYPE:** |  |  |
| Antidepressant (Yes vs. No) | 1.61 (1.15, 2.26) | 1.53 (1.05, 2.21) |
| Anxiolytic (Yes vs. No) | 1.17 (0.76, 1.82) | 1.06 (0.67, 1.69) |
| Sedative / Hypnotic (Yes vs. No) | 1.06 (0.71, 1.59) | 0.89 (0.58, 1.39) |
| **PATTERNS OF USE (DEFINITION 1):** |  |  |
| None | Ref. | Ref. |
| Antidepressant only | 1.53 (1.07, 2.19) | 1.43 (0.96, 2.12) |
| Anxiolytic only | 1.10 (0.68, 1.80) | 0.96 (0.57, 1.62) |
| Sedative / Hypnotic only | 0.95 (0.62, 1.45) | 0.82 (0.51, 1.30) |
| Antidepressant + Anxiolytic | 1.56 (0.50, 4.84) | 1.76 (0.53, 5.88) |
| Antidepressant + Sedative / Hypnotic | 4.67 (0.59, 36.96) | 3.32 (0.37, 30.12) |
| Anxiolytic + Sedative / Hypnotic | 1.29 (0.25, 6.71) | 0.88 (0.16, 4.88) |
| Antidepressant + Anxiolytic + Sedative / Hypnotic | -- | -- |
| **PATTERNS OF USE (DEFINITION 2):** |  |  |
| None | Ref. | Ref. |
| Antidepressant only | 1.53 (1.07, 2.19) | 0.70 (0.47, 1.04) |
| Anxiolytic only | 1.10 (0.68, 1.80) | 1.04 (0.62, 1.76) |
| Sedative / Hypnotic only | 0.95 (0.62, 1.45) | 1.22 (0.76, 1.96) |
| Combined | 2.00 (0.87, 4.60) | 0.55 (0.23, 1.35) |

^a^ Performance-based physical function was assessed using the Short Physical Performance Battery; ^b^ Adjusted for age (continuous), race, ethnicity, education, household income, marital status, smoking status, alcohol consumption, physical activity, body mass index (categorical), cardiovascular disease, hypertension, hyperlipidemia, diabetes, depressive symptoms (categorical), insomnia symptoms (categorical), and self-rated health, as described in tables 1 and 2.

**Table A.4.** Multiple linear regression models for relationships of antidepressant use at enrollment (1993—1998) with key continuous physical function outcomes after stratifying by level of depressive symptoms at enrollment (1993—1998)

|  | **Depressive symptoms ≤ 0.06** | | **Depressive symptoms > 0.06** | | **P _interaction_ *** |
| --- | --- | --- | --- | --- | --- |
| **Self-reported physical function at enrollment**  **[β (95% CI)]:** | N=2,329  -4.30 (-7.99, -0.61) | | N=2,228  -7.32 (-10.17, -4.48) | | 0.21 |
| **Performance-based physical function at Long-Life Study visit [β (95% CI)]:** | N=2,329  -0.36 (-1.01, 0.28) | | N=2,228  -0.34 (-0.76, 0.082) | | 0.95 |
| *Repeated measures of performance-based physical function:* |  | |  | |  |
| **Grip strength:** | N= 8,376 observations | | N=9,467 observations | |  |
|  | **F** | **P** | **F** | **P** | 0.70 |
| Medication | 0.93 | 0.33 | 5.54 | 0.018 |  |
| Medication x Visit | 0.09 | 0.96 | 0.25 | 0.86 |  |
|  |  |  |  |  |  |
| **Chair stand:** | N=7,951 observations | | N=8,832 observations | |  |
|  | **F** | **P** | **F** | **P** | 0.84 |
| Medication | 10.12 | 0.0015 | 22.42 | <0.0001 |  |
| Medication x Visit | 0.54 | 0.65 | 0.71 | 0.54 |  |
|  |  |  |  |  |  |
| **Gait speed:** | N=8,349 observations | | N=9,413 observations | |  |
|  | **F** | **P** | **F** | **P** | 0.57 |
| Medication | 2.53 | 0.11 | 1.99 | 0.15 |  |
| Medication x Visit | 1.45 | 0.23 | 0.10 | 0.96 |  |

* P value for the two-way interaction term between antidepressant use at enrollment (1993—1998) and depressive symptoms at enrollment (1993—1998) when included in a multivariable linear regression model that controls for sociodemographic, lifestyle, and health characteristics.

**Table A.5.** Multiple linear regression models for relationships of hypnotic use with continuous key physical function outcomes before and after stratifying by level of insomnia symptoms

|  | **Insomnia symptoms ≤ 9** | | **Insomnia symptoms > 9** | | **P _interaction_ *** |
| --- | --- | --- | --- | --- | --- |
| **Self-reported physical function at enrollment**  **[β (95% CI)]:** | N=3,504  -0.57 (-4.46, 3.30) | | N=1,053  -1.58 (-6.11, 2.96) | | 0.55 |
| **Performance-based physical function at Long-Life Study visit [β (95% CI)]:** | N=3,504  -0.01 (-0.66, 0.64) | | N=1,053  -0.15 (-0.81, 0.52) | | 0.69 |
| *Repeated measures of performance-based physical function:* |  | |  | |  |
| **Grip strength:** | N=13,155 observations | | N=4,688 observations | |  |
|  | **F** | **P** | **F** | **P** | 0.006 |
| Medication | 4.31 | 0.038 | 4.15 | 0.042 |  |
| Medication x Visit | 0.07 | 0.97 | 0.52 | 0.66 |  |
|  |  |  |  |  |  |
| **Chair stand:** | N=12,408 observations | | N=4,375 observations | |  |
|  | **F** | **P** | **F** | **P** | 0.15 |
| Medication | 3.49 | 0.062 | 0.17 | 0.68 |  |
| Medication x Visit | 1.52 | 0.21 | 0.15 | 0.93 |  |
|  |  |  |  |  |  |
| **Gait speed:** | N=13,117 observations | | N=4,645 observations | | 0.08 |
|  | **F** | **P** | **F** | **P** |  |
| Medication | 1.61 | 0.20 | 1.62 | 0.20 |  |
| Medication x Visit | 0.52 | 0.66 | 0.37 | 0.77 |  |

* P value for the two-way interaction term between hypnotic use at enrollment (1993—1998) and insomnia symptoms at enrollment (1993—1998) when included in a multivariable linear regression model that controls for sociodemographic, lifestyle, and health characteristics.
